# The costs of treating vaginal and vulval cancer in England (2009–2015)

**DOI:** 10.1186/s12889-020-08545-4

**Published:** 2020-04-06

**Authors:** Stephanie Stephens, Anuja Chatterjee, Victoria Coles, Robin Crawford

**Affiliations:** 1 Pharmerit International, York, UK; 2 Merck Sharp & Dohme Limited, Maidenhead, UK; 3 Addenbrookes Hospital, Cambridge University Hospitals NHS Foundation Trust, Cambridge, UK

**Keywords:** Human papillomavirus, Vaginal cancer, Vulval cancer, Retrospective, Resource use, Cost, England, Hospital Episode Statistics

## Abstract

**Background:**

Human papillomavirus (HPV) infection is a pre-requisite for cervical cancer, which represents the third most common cancer among women worldwide. A causal relationship also exists between HPV and cancer in other areas of the female reproductive system including the vagina and vulva. Whilst the incidence of vaginal cancer in the UK has remained relatively stable over the past 25 years, vulval cancer rates are increasing. A body of literature exists on the epidemiology and aetiology of vaginal and vulval cancer, but little is known about the economic burden. The objective of this study was to quantify the costs of treating these cancers on the National Health Service (NHS) in England.

**Methods:**

Inpatient and outpatient episodes were derived from Hospital Episode Statistics (HES). Health Resource Group (HRG) tariffs and National Reference Costs were used to estimate the cost of treating pre-cancerous and invasive vaginal and vulval lesions in England.

**Results:**

The study showed that for the 5 years from 2009/2010 to 2014/2015 the total cost associated with pre-cancerous and invasive vaginal and vulval lesions was over £14 million per year on average (95% of which was attributed to inpatient costs). Vulval cancer accounted for the largest proportion; an estimated 60% of the total cost (£8.82 million). On average 4316 patients per year in England were admitted to hospital and 912 patients attended outpatient settings for pre-cancerous and invasive disease of the vagina and vulva.

**Conclusion:**

The results indicate that vaginal and vulval cancer cost the English health care system over £14 million per year. Given the causal role of HPV in a proportion of these cancers, preventative measures such as the national HPV immunisation programme have the potential to reduce the economic burden. To ensure optimal use of NHS resources, it is important that future economic evaluations of such preventative measures consider the full burden of HPV related disease.

## Background

Human papillomavirus (HPV) is one of the most prevalent sexually transmitted diseases worldwide with over 100 strains currently recognised, many of which are spread through sexual contact [1].

It is estimated that 75 to 80% of sexually active individuals will become infected with HPV in their lifetime [2, 3]. Reports suggest that sexually active women under 25 years are most at risk of HPV infection. The risk of HPV infection increases with multiple sexual partners and with lower age at first intercourse. Most infections are harmless and cleared by the immune system, usually within 24 months, but persistent infection can be carcinogenic. HPV is a fundamental cause of cervical cancer and is aetiologically associated with cancers at other sites, including the vagina and vulva. Current data suggest that HPV infection may be associated with approximately 25% of vulval cancers and approximately 78% of vaginal cancers globally [4]. As with other ano-genital cancers, the susceptibility to vaginal and vulval cancer also increases with age [5–7]. While the age-standardised incidence of vaginal cancer has remained relatively stable over the past 25 years, vulval cancer is on the rise, with a 12% increase in age-standardised incidence rates seen between 2004 and 2006 and 2014–2016 [5, 6].

Treatment of vaginal and vulval cancers involves surgical intervention - ranging from ablation and local excisions to vaginectomy and vulvectomy - as well as radiotherapy and chemotherapy. The costs of treating vaginal and vulval cancers in England are poorly understood. Published economic evaluations of HPV immunisation use estimations based on the cost of cervical cancer or non-cervical cancers in other countries [8–11]. Thus, at present there is an unmet need in terms of published estimates of the costs associated specifically with vaginal and vulval cancers in England.

The objective of this study is to provide an update of the current inpatient and outpatient activity related to these cancers and concurrently estimate the healthcare costs associated with treatment.

## Methods

To estimate the costs of treating vaginal and vulval cancers from an English healthcare perspective, a retrospective (non-comparative) case series analysis was conducted using patient level data extracted from the Hospital Episode Statistics (HES) database. HES includes records of all care funded by the English NHS (all admissions, accident & emergency attendances and outpatient appointments at NHS hospitals in England), allowing estimation of the secondary care treatment costs associated with pre-cancerous and invasive vaginal and vulval cancer lesions in England. More information on the patient population included in the HES database are available from the NHS digital website [12]. Based on advice from a clinician specializing in gynaecological oncology, admitted patient care (APC) (inpatient) records were identified based on the presence of primary, secondary or tertiary diagnosis of the following ICD-10 codes: C52 – vaginal cancer, C51 – vulval cancer, D072 and N89 – vaginal dysplasia, D071 and N90 – vulval dysplasia. Data on outpatient attendances were extracted for the same list of ICD-10 codes but were confined to records with a primary or secondary diagnosis only, reflecting the more disease specific nature of post-treatment care.

In accordance with the National Tariff/Payment by Results (PbR) policy, the annual management costs for pre-invasive and invasive vaginal and vulval lesions were estimated from the health payers’ perspective [13].

### HES data collection

All finished consultant episodes (FCE) and outpatient attendances related to one of the selected ICD-10 codes were extracted from the HES database along with anonymised patient identification numbers to estimate the annual number of hospitalised patients. Records were extracted between the data years of 2009/10 and 2014/15. The time frame for analysis was restricted to these data years because these were the latest full-year data available from HES at the time of data request. No other inclusion or exclusion criteria were applied.

### Data aggregation and costing

The number of patients undergoing treatment for vaginal or vulval cancer in each year of the study period was determined by tracing the unique patient identifiers (HESID) assigned to each FCE. Mean annual patient numbers were then calculated. NHS funded healthcare providers in England are reimbursed under the Payment by Results (PbR) scheme [14]. The currencies for payment under PbR are healthcare resource groups (HRG). Each year, payment tariffs for each HRG are determined using retrospective analysis of costing data submitted from previous years. In order to derive relevant HRGs for care delivered to vaginal and vulval cancer patients, inpatient FCEs were aggregated into spells of care (from hospital admission to discharge) using software publicly available from the NHS [15]. Although most spells comprise a single FCE under the care of one consultant from admission to discharge, in some cases spells are spread across several FCEs. A spell is also a more robust activity measure than an FCE as the latter can be easily influenced (e.g., by transferring patients between consultants). The 2016/17 local payment grouper was used to group FCEs into spells of care and derive a single Healthcare Resource Group (HRG) [15]. Core HRGs were then cross-referenced with the National Tariff 2016/17 (reflecting 2016 costs) to estimate the associated spell cost. Costing analyses were performed in Microsoft Excel 2016. Standard deviations were computed in Microsoft Excel using the 2009–2014 total cost numbers and were calculated using standard methods, i.e., by taking the square root of the variance, the variance being the average of the squared difference of each total cost per year subtracted from the mean total cost over the 2009/2010–2014/2015 time frame.

The hospital cost per patient was calculated by first calculating the total HRG costs for each spell recorded in the data across all patients per year. Following this the average hospital cost per patient was calculated by dividing the total HRG costs in a given year by the annual number of hospitalised patients in that specific year. For inpatients, the costs associated with the spell of care included all costs associated with the initial diagnosis (if this took place in a hospital), surgical procedures and hospital-based medical treatments. Outpatient costs were estimated by grouping consultations by treatment specialty based on Treatment Function Codes (TFCs) and whether the consultation was the first of a series or a follow-up. As with the inpatient data, all activities for which reimbursement rates are locally negotiated, such as outpatient chemotherapy and radiotherapy sessions, were disaggregated from the core HRG. For specific types of therapy, including but not limited to chemotherapy, radiotherapy and rehabilitation, costs are not included within the National Tariff due to wide regional variations in fees and practice. For such therapies, the HRGs derived from the HES data were cross-referenced with the 2016 National Reference Costs [16].

Each data extract was validated and cleaned prior to delivery by NHS Digital [12, 17]. A second cleaning process was conducted, checking for duplicates and missing fields that may impede the grouping process. Any data that could not be matched with the grouper was excluded from the costing analysis. Data was processed using Microsoft Excel 2016.

Some FCEs could not be grouped due to inaccurate coding or missing codes within data fields. Because of missing codes, three types of error were mostly seen. These were FCE error counts, spell error counts and the exclusion of errors and missing data in the age field. In these cases (ranging between 0.36% of records for vaginal dysplasia and 1.61% of records for vaginal cancer), a ‘UZ01Z’ HRG code was generated, which is defined as ‘Data invalid for grouping’. Table 1 shows the number of errors seen, and therefore the number of records excluded from the analysis at the grouper stage, stratified by ICD-10 code per year. We did not impute any values. The grouper carried out the exclusions in a systematic manner.

**Table 1:** Missing values as identified from the HRG Grouper per ICD-10 code per year

ICD-10	Description	Year	FCE Error Count	Spell Error Count
C51	Vulval Cancer	2009/10	83	59
		2010/11	76	52
		2011/12	104	84
		2012/13	32	14
		2013/14	20	7
		2014/15	22	8
N90	Vulval Dysplasia	2009/10	30	3
		2010/11	39	4
		2011/12	43	7
		2012/13	49	2
		2013/14	53	5
		2014/15	51	4
D071	Vulval Dysplasia	2009/10	34	8
		2010/11	57	8
		2011/12	53	8
		2012/13	52	1
		2013/14	56	2
		2014/15	59	6
C52X	Vaginal Cancer	2009/10	20	14
		2010/11	15	12
		2011/12	95	60
		2012/13	2	2
		2013/14	31	28
		2014/15	22	21
N89	Vaginal Dysplasia	2009/10	18	1
		2010/11	14	1
		2011/12	20	1
		2012/13	32	1
		2013/14	29	1
		2014/15	23	1
D072	Vaginal Dysplasia	2009/10	12	2
		2010/11	13	2
		2011/12	20	7
		2012/13	10	1
		2013/14	17	1
		2014/15	10	2

Missing data is a result of poor clinical coding in the initial phase of data collection and cannot be rectified. This is a limitation of the current analyses which could lead to potential underestimations of the true costs, however, where possible all records (regardless of missing data) were included in the patient demographic analysis.

## Results

### Study sample overview

Between 2009/10 and 2014/15, an average of 4316 patients were admitted to hospital each year in England for vaginal or vulval related cancer or dysplasia. The most frequent diagnoses were related to diseases of the vulva (vulval dysplasia mean inpatients = 1726, and vulval cancer mean inpatients = 1621). On average, age at diagnosis was higher for invasive cancer cases than for dysplasia cases. Furthermore, across all lesion types, patients presenting with diseases of the vulva were older on average than those with vaginal disease, with a mean age at diagnosis ranging from 49 for vaginal dysplasia to 68 for vulval cancer (Table 2). The data shows a trend towards an increasing number of inpatients admitted for vulval dysplasia over time, with a 11% increase from 2009/10 to 2014/15 (Table 2). This mirrors published reports on the increasing incidence of vulval cancers in the UK [5, 6].

**Table 2:** Incidence of Vaginal and Vulval Cancer Over Time

	2004	2005	2006	2007	2008	2009	2010	2011	2012	2013	2014	2015	2016
Vaginal Cancer													
Inpatient Numbers (HES data)	–	–	–	–	–	428	436	460	425	453	427	–	–
Outpatient Numbers (HES data)	–	–	–	–	–	82	78	127	71	126	158	–	–
Age-Standardised Incidence Rate per 100,000 (Cancer Research Statistics)	0.8	0.9	0.9	0.8	0.8	0.9	0.9	0.9	0.8	0.8	0.8	0.7	0.8
Vulval Cancer													
Inpatient Numbers (HES data)	–	–	–	–	–	1577	1560	1591	1736	1644	1619	–	–
Outpatient Numbers (HES data)	–	–	–	–	–	199	193	282	376	434	612		
Age-Standardised Incidence Rate per 100,000 (Cancer Research Statistics)	3.5	3.5	3.7	3.8	3.9	4.0	3.9	3.9	4.1	4.2	4.0	4.1	3.9

### Outpatient attendances

For the diseases of interest over the period studied 912 patients on average attended outpatient facilities each year. The most frequent diagnoses were vulval cancer (mean patients = 349), and vaginal dysplasia (mean patients = 261) (Table 3). The number of patients receiving outpatient treatment for vaginal dysplasia suddenly and disproportionately increased from 2012/13 (93) to 2013/14 (681).

**Table 3:** Inpatient and outpatient admission statistics

	Mean Age at Diagnosis in Years	Mean Annual Inpatients	Annual Inpatient Cost per Patient (SD)	Mean Annual Outpatients	Annual Outpatient Cost per Patient (SD)	Total Annual Cost of Disease (SD)
Vaginal Cancer	61.2	438	£5335 (£176)	107	£1850 (£536)	**£2,535,631 (£84,825)**
Vulval Cancer	68.5	1621	£5173 (£386)	349	£1231 (£166)	**£8,816,813 (£553,570)**
Vaginal Dysplasia	49.5	531	£1250 (£96)	261	£401 (£102)	**£767,634 (£113,141)**
Vulval Dysplasia	54.3	1726	£1487 (£31)	195	£228 (£29)	**£2,609,937 (£113,442)**
**Total**		**4316**		**912**		**£14,730,015 (£478,362)**

*(SD Standard deviation); Total numbers are provided in****Bold***

### Hospital spells

On average, 7077 hospital spells (across 4316 patients who experienced at least one hospital spell) and 3834 outpatient attendances (across 912 patients who recorded an outpatient visit) each year were recorded across all conditions studied. Annually, the average vaginal cancer inpatient had 3 hospital spells and the average vaginal cancer outpatient had 9 outpatient attendances. This was lower on average for vulval cancer patients (2 hospital spells / 7 outpatient attendances per year). Dysplasia patients had fewer hospital visits (1 hospital spell and 1 outpatient attendance per year on average). Mean spell durations were highest for invasive vulval cancer (5 days) and invasive vaginal cancer (3 days). Ranging from 82% for invasive cancers to 97–98% for dysplasia, most hospital spells were elective admissions. Elective day case admissions were observed in 42% of vaginal cancer, 26% of vulval cancer, 73% of vaginal dysplasia, and 67% of vulval dysplasia hospital spells. Excess bed days were observed in 10% of vulval cancer hospital spells and in ≤3% of spells for all other conditions studied.

### Treatment costs

**Fig. 1:**
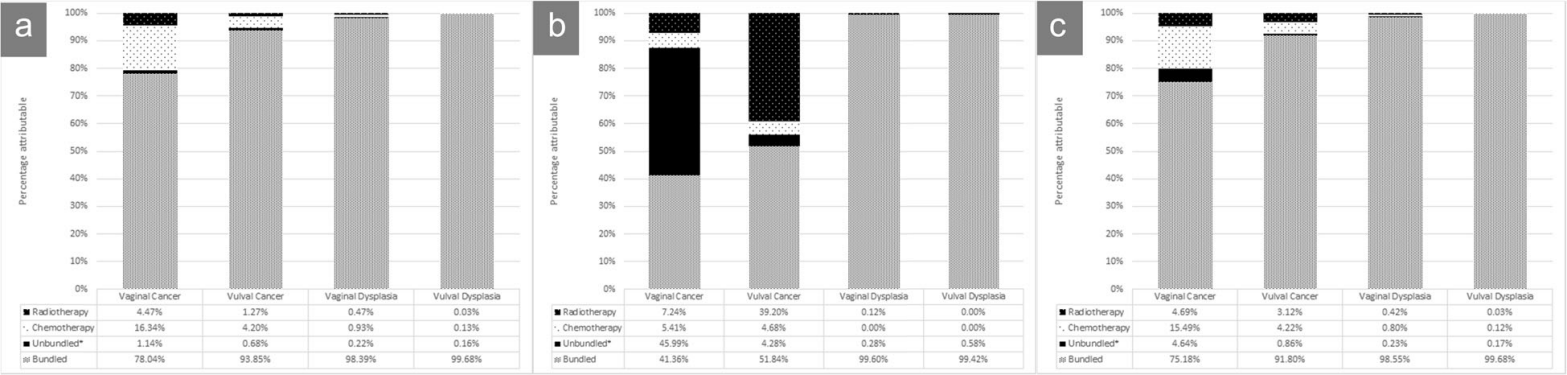
Mean annual cost across genital cancer and dysplasia per type of care. *Excludes radiotherapy and chemotherapy unbundled costs **a** Inpatient costs **b** Outpatient costs **c** Total costs

The mean annual cost per patient (setting dependent) is provided in Table 3. Mean annual inpatient costs were highest for the invasive cancers, at £5335 for vaginal cancer and £5173 for vulval cancer. Dysplasia were associated with lower inpatient costs of between £1250 and £1487 on average per year. The same pattern was seen for outpatients, where costs associated with invasive cancer patients were £1850 for vaginal cancer and £1231 for vulval cancer, and those associated with dysplasia ranged from £228 to £401 on average per year. Across all diseases and sites, 95% of costs were attributable to inpatients (£13.95 million vs £777,000). Table 4 provides a breakdown of the mean annual inpatient and outpatient cost per type of cost category. In PbR, bundled costs include all care received in a hospital setting except unbundled HRGs such as chemotherapy, radiotherapy, rehabilitation, palliative care, specific diagnostic imaging and high cost drugs. Unbundled HRGs were introduced by PbR to make it possible to separately report, cost and remunerate these different components within a care pathway away from the traditional hospital setting. Chemotherapy and radiotherapy have been disaggregated from other unbundled costs for the purposes of this research. Across all conditions studied, 9% of costs were attributable to unbundled costs, but this varied by disease and site (Table 4, Fig. 1). On average, the proportion of total costs attributed to unbundled elements of care, including chemotherapy and radiotherapy, is highest in invasive cancers (24.8% in vaginal cancer and 18.2% in vulval cancer). Radiotherapy is a key treatment option in vulval cancer patients, with 39.2% of all outpatient treatment costs being attributed to such therapy. In vaginal cancer patients, almost half of all outpatient treatment costs are due to rehabilitation (including physical rehabilitation, psychological support, and provision of information) and palliative care, demonstrating the sometimes lengthy and intense post-surgery care these patients can experience. As would be expected, a very small proportion of the total cost associated with pre-cancers was attributable to unbundled costs. Across all disease sites, chemotherapy is associated with the highest cost burden when looking at all unbundled elements of care.

**Table 4:** Mean annual cost per genital cancer and dysplasia per type of care

Inpatient					
Disease of Interest	Bundled (SD)	Unbundled* (SD)	Chemotherapy (SD)	Radiotherapy (SD)	Total (SD)
Vaginal Cancer	£1,824,341 (£124,012)	£26,654 (£3028)	£382,057 (£55,121)	£104,575 (£5412)	**£2,337,628 (£104,567)**
Vulval Cancer	£7,871,209 (£618,739)	£57,153 (£10,785)	£352,365 (£59,023)	£106,098 (£17,420)	**£8,386,826 (£612,849)**
Vaginal Dysplasia	£652,098 (£51,321)	£1436 (£1114)	£6156 (£6927)	£3107 (£3810)	**£662,798 (£54,641)**
Vulval Dysplasia	£2,557,355 (£134,342)	£4194 (£2453)	£3262 (£2603)	£705 (£1226)	**£2,565,516 (£131,438)**
**Total**	**£12,905,004 (£591,585)**	**£89,438 (£11,367)**	**£743,840 (£105,324)**	**£214,486 (£17,401)**	**£13,952,767 (£573,092)**
Outpatient					
Vaginal Cancer	£81,897 (£37,748)	£91,068 (£23,366)	£10,703 (£8501)	£14,334 (£9140)	**£198,003 (£69,410)**
Vulval Cancer	£222,895 (£132,128)	£18,424 (£9171)	£20,109 (£17,461)	£168,558 (£63,196)	**£429,987 (£215,690)**
Vaginal Dysplasia	£104,414 (£140,277)	£298 (£201)	£0 (£0)	£124 (£304)	**£104,836 (£140,185)**
Vulval Dysplasia	£44,162 (£30,812)	£259 (£245)	£0 (£0)	£0 (£0)	**£44,421 (£32,252)**
**Total**	**£453,369 (£283,653)**	**£110,049 (£27,717)**	**£30,813 (£23,850)**	**£183,017 (£71,720)**	**£777,247 (£390,837)**
**Total (Inpatient + Outpatient)**					
Vaginal Cancer	**₤1,906,239 (£101,890)**	**₤117,722 (£24,323)**	**₤392,761 (£52,928)**	**₤118,909 (£13,365)**	**₤2,535,631 (£84,825)**
Vulval Cancer	**₤8,094,105 (£572,787)**	**₤75,578 (£17,945)**	**₤372,474 (£63,309)**	**₤274,657 (£60,135)**	**₤8,816,813 (£553,570)**
Vaginal Dysplasia	**₤756,513 (£127,879)**	**₤1734 (£1212)**	**₤6156 (£5286)**	**₤3232 (£1146)**	**₤767,634 (£113,141)**
Vulval Dysplasia	**₤2,601,517 (£115,984)**	**₤4453 (£2390)**	**₤3262 (£2603)**	**₤705 (£1226)**	**₤2,609,937 (£113,442)**
**Total**	**₤13,358,373 (£3,259,701)**	**₤199,487 (£56,700)**	**₤774,652 (£218,346)**	**₤397,502 (£129,209)**	**₤14,730,015 (£478,362)**

*Excludes radiotherapy and chemotherapy unbundled costs; (SD Standard deviation); Total numbers are provided in***Bold***

Vaginal dysplasia has the lowest total cost burden. This is due to the low patient numbers recorded with this disease. Vulval cancer places the highest total burden on NHS resources.

Figure 1 illustrates the proportion of costs attributable to bundled, chemotherapy, radiotherapy, and other unbundled costs for each disease state, looking at inpatient costs (Fig. 1a), outpatient costs (Fig. 1b), and total cost burden (Fig. 1c).

## Discussion

This analysis was conducted to investigate the annual number of patients seen for outpatient and inpatient care with pre-cancerous and invasive vaginal and vulval cancer lesions in England and their associated management costs. Data were analysed from 2009/10 to 2014/15, representing the most recent patient records available at the time of data delivery to the authors.

The study showed that between 2009/10 and 2014/15, the total cost of pre-cancerous and invasive vaginal and vulval cancer lesions in English secondary care amounted to over £14 million per year, with an average of 4316 patients hospitalised for these conditions and 7077 inpatient spells of care recorded. In addition, approximately 912 patients attended an outpatient facility each year. Diagnoses relating to the vulva were most frequent, with on average, 38% of patients diagnosed with vulval cancer and 36% with vulval dysplasia. Vulval cancer also accounted for the largest proportion of overall costs (60%), at £8.82 million on average per year. The data further showed an increasing trend in the number of inpatients admitted for vulval dysplasia over time, with an 11% increase seen from 2009/10 to 2014/15. Improved diagnostic techniques, lifestyle changes and an increase in the life expectancy of women over time may all contribute to this increase. Regardless of the cause, an increased disease incidence is a concern and should be addressed.

Our analyses show that the number of patients receiving outpatient treatment for vaginal dysplasia suddenly and disproportionately increased from 2012/13 to 2013/14. HPV testing was introduced as part of the NHS Cervical Screening Programme for England in April 2011. HPV testing is provided to triage women whose first cytology sample shows borderline nuclear changes or low grade dyskaryosis and for test of cure at 6 months for newly treated women with negative, borderline or low grade dyskaryosis. Those women who are positive for high risk HPV DNA in either the triage group or test of cure, including women with negative cytology in the test of cure group, are referred for further colposcopy. According to the British Society for Colposcopy and Cervical Pathology, some colposcopy services experienced an increase in referrals for cervical cancer following the introduction of HPV testing [18]. This, combined with the increase in outpatient cases seen in the data between 2012 and 2014, raises the question of whether the use of HPV testing in the low-grade setting may have also led to more cases of vaginal dysplasia being detected.

When comparing our results to the estimates available on a European and global scale, the prevalence and economic burden of vulval and vaginal cancers have primarily been estimated in the context of cervical cancers and HPV-attributable anogenital cancers [19–21]. The latest global prevalence figures. report 8500 and 12,000 cases of HPV-related vulval and vaginal cancer [19]. The standalone evidence on the national-level burden of vulval and vaginal cancers are rare [22] especially within the English healthcare context [23, 24]. Recent, comparable primary evidence using similar national-level data from Scottish [22], French [23, 25], and Danish [26] perspectives has been published; although, with the exception of Abramowitz et al. [25], the breakdown of radio- and chemo-therapy costs have not been stratified in these publications. Abramowitz et al. reported a higher proportion of chemotherapy and radiotherapy treatments and consequent per patient cost of these treatments in vaginal cancer cases than in vulval cancer cases. This was also seen, to a greater extent, in the current study (3.9-fold increase in per patient costs of chemotherapy and radiotherapy in vaginal cancer cases versus vulval cancer cases in the current study compared with a 1.8-fold increase seen in Abramowitz et al). Consistent with our study, all these European publications found the per patient cost of treating vaginal cancers to be higher than vulval cancer. There is some variation in the way results have been presented across studies, for example Olsen et al. 2012 report costs in terms of years since diagnosis, where the first-year treatment costs were considerably higher than the second-year costs for treating both cancers [26]. Wakeham and Kavanagh (2014) [22] do not present specific cost estimates but rather review the epidemiology of disease [22]. Borget et al. present cost results in a similar way to the current paper. They find the costs of vulval cancers in France to be approximately 40% higher than for vaginal cancers [23]. Our findings indicate a much larger difference in overall costs when looking at data from England (approximately 250% higher for vulval cancers). This may in part be due to a limitation noted in the Borget study where it is stated that the costs have been underestimated and estimates of outpatient and daily allowance costs have been based on several assumptions. Amongst other publications using secondary evidence sources [19, 24, 27], a recent UK modelling study [28] also attempted to estimate the 20-year lifetime treatment-cost of vulval or vaginal cancer (£13,650 per case (2011 costs)) [27]. In summary, the last national level database analysis reporting costs of these cancers was carried out in 2009 [21, 23, 26], with the latest published prevalence figures available up till 2011 [4, 22]. Therefore, our study adds the most up-to-date costs and burden of managing vulval or vaginal cancers, using longitudinal data from an English perspective, to this scarcely researched body of evidence.

### Study limitations

This analysis is not without limitations. Firstly, we only included patients with a relevant primary, secondary or tertiary diagnosis code in the analyses. Restricting the presence of an ICD-10 code in this way may have introduced some selection bias. However, including further diagnoses may have resulted in costs not related to vaginal and vulval cancer being included in the analysis. Secondly, although it was possible to ascertain whether a patient had been admitted to hospital multiple times within the data years extracted, it was not possible to distinguish between an initial or recurrent patient. Thus, the estimated mean annual cost per patient reflects the average costs of all cases incurring costs within that year, regardless of the timing of their diagnosis. Furthermore, as ICD-10 codes are the basis for which a diagnosis is coded, it was not possible to determine the stage of cancer at the time of admission. A higher stage would likely translate to higher costs and resource use. For invasive cancers, it is reasonable to expect that the number of inpatients is an accurate reflection of the total patient numbers, since very few vaginal or vulval cancer patients will go through their entire treatment pathway as an outpatient, but this may not hold true for patients with dysplasia where much more treatment is provided in the outpatient setting. Furthermore, patients could not be traced over time and therefore it was not possible to determine how many patients had a hospital event recorded in more than one data year. Consequently, estimations for the total cost per patient for the entire duration of their treatment could not be calculated. It was also not possible to segregate HPV-positive cancer cases from HPV-negative cancer cases in the current analyses. Rasmussen et al. have shown that patients with a HPV-positive vulval cancer diagnosis generally have a more optimistic prognosis than patients who are diagnosed with HPV-negative vulval cancer [29]. This may mean that the costs of treating HPV positive cases differ to those for HPV negative cases. Further investigation to determine if there is a difference in costs is needed. Despite these limitations, the present analysis shows that there is a cost burden of over £14 million per year associated with the treatment of vaginal and vulval cancers in England, in addition to the physical (e.g. pain, fatigue, bowel / urinary difficulties), emotional (e.g. anxiety, fear, depression) and personal (e.g. returning to work, adjustments to sex life, (new) relationships) burden felt by the patients themselves and their caregivers [30].

The present study provides results which inform the Department of Health and other decision makers about the costs of vaginal and vulval cancers, consequently helping them to realise the value of thorough testing for female genital cancers, as well as endorsing the WHO ambition of HPV elimination. A recent study in Australia has suggested that over the next 15 to 20 years, we will see declines in cervical cancer incidence and mortality rates due to such screening which will in turn reduce healthcare related costs [31]. Proper implementation of HPV immunization programs could increase this positive impact further as their benefit is not only limited to reducing cervical cancer cases because they also protect against other anogenital cancers, such as vaginal and vulval lesions.

## Conclusion

In conclusion, patients with vaginal and vulval cancers cost the NHS in England over £14 million per year. Given the association of HPV with approximately 25% of vulval cancer and 78% of vaginal cancer cases, preventative measures such as the national HPV immunisation programme have the potential to reduce the cost burden on the healthcare system. Taken together with our previous studies on the costs of anal, penile and head and neck cancer, this demonstrates the significant – and rising - burden of HPV-related disease on the health system and highlights the importance of acknowledging that HPV vaccination has the potential to prevent disease beyond cervical cancer. To ensure optimal use of NHS resources, it is important that future economic evaluations of such preventative measures consider the full burden of HPV related disease.

## Data Availability

The data that support the findings of this study are available from NHS Digital, but restrictions apply to the availability of these data, which were used under license for the current study, and so are not publicly available.

## Abbreviations

APC: Admitted patient care
FCE: Finished consultant episodes
HES: Hospital Episode Statistics
HPV: Human papillomavirus
HRG: Health Resource Group
NHS: National Health Service
PbR: Payment by Results
SD: Standard deviation
